# Expanding trauma education during war: pediatric trauma fundamentals training in Ukraine

**DOI:** 10.3389/fpubh.2024.1448075

**Published:** 2024-09-06

**Authors:** David Mills, Alexis Schmid, David Lewander, Michelle Gonnet, Oleksii Lopatniuk, Oleksandra Demetska, Olena Sorokina, Anna Bolonska, Ramona Sunderwirth, Sean Kivlehan, Kathleen Murray, Michelle Niescierenko

**Affiliations:** ^1^Department of Pediatrics, School of Medicine, University of California San Diego, La Jolla, CA, United States; ^2^Global Health Program, Boston Children’s Hospital, Boston, MA, United States; ^3^Harvard Humanitarian Initiative, Cambridge, MA, United States; ^4^International Medical Corps, Los Angeles, CA, United States; ^5^Dnipro State Medical University, Dnipro, Ukraine; ^6^Icahn School of Medicine at Mount Sinai, New York, NY, United States; ^7^Department of Emergency Medicine, Brigham and Women’s Hospital, Boston, MA, United States; ^8^Harvard Medical School, Boston, MA, United States

**Keywords:** war and conflict, Ukraine, pediatric trauma, emergency care, education

## Abstract

**Introduction:**

On 24 February 2022, Russia launched a large-scale offensive in Ukraine, resulting in significant casualties to civilians, including children. As part of a seven-stream trauma education initiative, a novel pediatric trauma fundamentals course (PTF) was developed to provide standalone pediatric trauma education by our academic/NGO partnership. The objective of the program was to develop, implement, and evaluate a novel PTF educational course in the active armed conflict zone of Ukraine.

**Methods:**

A novel two-day PTF course was internally developed, translated into Ukrainian, and implemented across eight Oblasts (regions) in Ukraine from November 2022 to December 2023. Participants completed pre-and post-assessments in knowledge and self-confidence, and critical skills were assessed against objective skill checklists. Change in knowledge and self-confidence were analyzed, respectively, with the nonparametric Wilcoxon matched-pairs signed-rank test and McNemar’s test for paired data. Anonymous course evaluations were solicited after each course. Six to eight-week follow-up surveys were conducted to assess skill utilization and stewardship.

**Results:**

Four hundred and forty-six Ukrainian health care providers were trained during 30 courses across 8 Oblasts in Ukraine during the intervention period. Aggregated knowledge and self-confidence significantly improved across all measures. Ukrainian instructors of courses received higher raw scores across all evaluation points on instructor feedback surveys as compared to international instructors. Six to eight-week follow-up surveys demonstrated participants had positive views of the training, have used the training on patients, and have taught the material to other health care providers.

**Discussion:**

Our novel PTF intervention demonstrates a successful partnership-based model for implementing pediatric trauma education in an active conflict zone in Ukraine. Challenges to implementing such programs can be mitigated through strategic partnership-based models between academic institutions and organizations with local knowledge and expertise. Ukrainian instructors provide course experiences similar or superior to international instructors, likely due to multiple factors related to language, culture, and context.

## Background

On 24 February 2022, Russia expanded its war in Ukraine by launching a large-scale offensive across the country. Over the past 2 years, the conflict has devastated communities in Ukraine, leading to over 10,500 civilian deaths and almost 20,000 injured (1). This includes over 600 children killed and 1,350 injured (2). The ongoing conflict has caused immediate casualties while also leading to a significant and profound impact on public health and hospital infrastructure. Over 1,700 attacks on Ukraine’s health system have led to numerous medical facilities being damaged or destroyed, hundreds of health care workers killed (3), and significant disruptions of critical utilities supporting hospital functionality, including energy and water supply systems (4, 5). The prolonged nature of the conflict has also led to a critical shortage in health and medical supplies, overburdened health care workers, public health emergencies, and a significant mental health burden on the Ukrainian civilian population.

The escalation of the conflict has increased the need for trauma and emergency care throughout Ukraine, which has seen an exponential increase in war-related injuries from mechanisms such as penetrating trauma, burns, crush and blast injuries (6). The health consequence of these mechanisms of injury include complex traumatic injuries requiring immediate stabilization, advanced surgical interventions, rehabilitation, and comprehensive long-term care (6). In addition to the profound effect on hospitals, clinics, and medical supply chains, medical providers have faced unprecedented challenges to providing care, including the disruption of medical education and the transition of currently practicing providers toward trauma-based care and education.

The Harvard Humanitarian Initiative (HHI), a university-based interfaculty initiative, has partnered with organizations, agencies, and ministries of health to support humanitarian responses around the world (7). Building from prior relationships delivering Basic Emergency Care and Chemical, Biological, Radiological, Nuclear, and Explosives (CBRNE) courses in Ukraine after Russia’s annexation of Crimea in 2014 (8–10), HHI undertook a rapid needs assessment shortly after the 2022 Russian invasion to understand the trauma-related education required to meet the acute care needs across the country. HHI, in partnership with the International Medical Corps, developed a multi-stream trauma training initiative to provide Ukrainian health care workers, public safety officials, and civilians with training in trauma management (11). After a successful preliminary implementation of the multi-stream intervention and in response to feedback, a stand-alone pediatrics trauma course was developed to implement both at the country’s freestanding childrens’ hospitals and in general hospitals that receive pediatric patients.

Given that the participants of the course would either have a strong background in pediatrics or basic knowledge in adult trauma care with minimal pediatrics knowledge, we undertook the development of a Pediatric Trauma Fundamentals (PTF) course. We developed this course to fill the gap in pediatric-focused trauma education in wartime or conflict settings, while also ensuring a highly contextually appropriate curriculum tailored to the Ukrainian context. The two-day trauma training curriculum was planned and developed with input and feedback from Ukrainian partners. The overall objectives of the program included: (1) the development and implementation of a pediatric trauma fundamentals course to provide immediate training for health care providers in Ukraine and (2) the sustainable integration of the program into Ukrainian educational initiatives. The team sought to assess the effectiveness of the PTF educational implementation program on trauma theoretical knowledge, skills, and practical implementation during an active armed conflict in Ukraine.

## Methods

In the Summer of 2022, prior to the conclusion of the first phase of an overarching multi-stream trauma program in Ukraine, a general consensus between partner organizations (International Medical Corps and HHI) was made that a pediatric-specific trauma fundamentals course should be added as a stand-alone trauma education stream. A majority of pediatric trauma care takes place in a network of Ministry of Health-run freestanding childrens hospitals not previously targeted in the first phase of the multi-stream trauma program. Additionally, frontline regions with general hospitals that receive pediatric patients, in cities such as Mykolaiv, were included in the PTF trauma initiative.

### Course curriculum

A novel two-day course entitled ‘Pediatric Trauma Fundamentals’ was conceptualized and developed internally by a core group of seven pediatric emergency medicine physicians and nurses. The core team had previous experience in curriculum development and had extensive experience in the humanitarian sector through engagement with various academic and international agencies and organizations. Content was sourced from several international resources including the World Health Organization (WHO) and the International Committee of the Red Cross Basic Emergency Care, Advanced Trauma Life Support, UpToDate, Fleisher and Ludwig’s Textbook of Pediatric Emergency Medicine, and the Boston Children’s Global Health Program pediatric resuscitation course (12). Curriculum topics & modules are listed in Table 1.

**Table 1:** PTF curriculum modules.

Pediatric trauma fundamentals: curriculum modules
Introduction to pediatric trauma
Airway and breathing
Circulation
Head trauma
Thoracic trauma
Abdominal and pelvic trauma
Spine/spinal cord trauma
Burns
Blast injuries
Chemical injuries
Principles of teamwork

Educational delivery modalities included didactic frontal lectures, hands-on skills stations, interactive case discussions, and team-based simulation scenarios. All materials were translated into Ukrainian and reviewed by International Medical Corps interpreters based in Ukraine for language, context and culturally specific considerations. The two-day schedule was adapted as needed for safety/security considerations, which included tailoring course start and end times based on travel requirements and daily safety/security briefs providing real time information about impending attacks. Supplementary PTF videos were developed for high-yield topics in pediatric trauma. Links were provided for students during the course and made publicly available on YouTube (13).

A second curriculum was developed for the “training of trainers” (ToT) component of the course. In addition to the two-day PTF course, three additional days for a five-day course included one day on adult learning and teaching theory and two days on flipped classroom, student-driven didactic, skills and simulation practice.

### Course delivery

Courses were delivered in person by international English-speaking instructors and Ukrainian instructors in Ukrainian language, both with live Ukrainian/English bi-directional translation for the duration of all courses. The PTF course was implemented in a three-part approach: (1) international instructor led PTF courses, (2) ToT courses which developed a cohort of Ukrainian instructors to teach PTF to Ukrainian participants, and (3) Ukrainian instructor-led courses with international mentorship. During the initial implementation of the PTF intervention (November 2022 to April 2023), English speaking international instructors provided in person instruction to Ukrainian participants. International instructors were recruited by HHI and International Medical Corps. Given the safety and security considerations in an active conflict zone, and to limit the number of unique providers teaching courses, international instructors were obligated to dedicate two-week blocks of course delivery during the PTF intervention. Recruitment for Ukrainian learner participants was undertaken by International Medical Corps and sought out the following priority medical providers as course participants: pediatricians, pediatric surgeons (all specialties), general practitioners, general surgeons, and any other provider who cares for traumatically injured children. During part two of the PTF implementation (August 2023 to December 2023), Ukrainian participants were identified to attend a five-day ToT course to become instructors. Ukrainian instructors were identified by recommendation from Ukrainian host universities. ToT courses were taught by in person international instructors. PTF courses thereafter were led by Ukrainian instructors with international instructors onsite to provide active mentorship, content expertise and educational delivery feedback.

### Training sites

**Figure 1:**
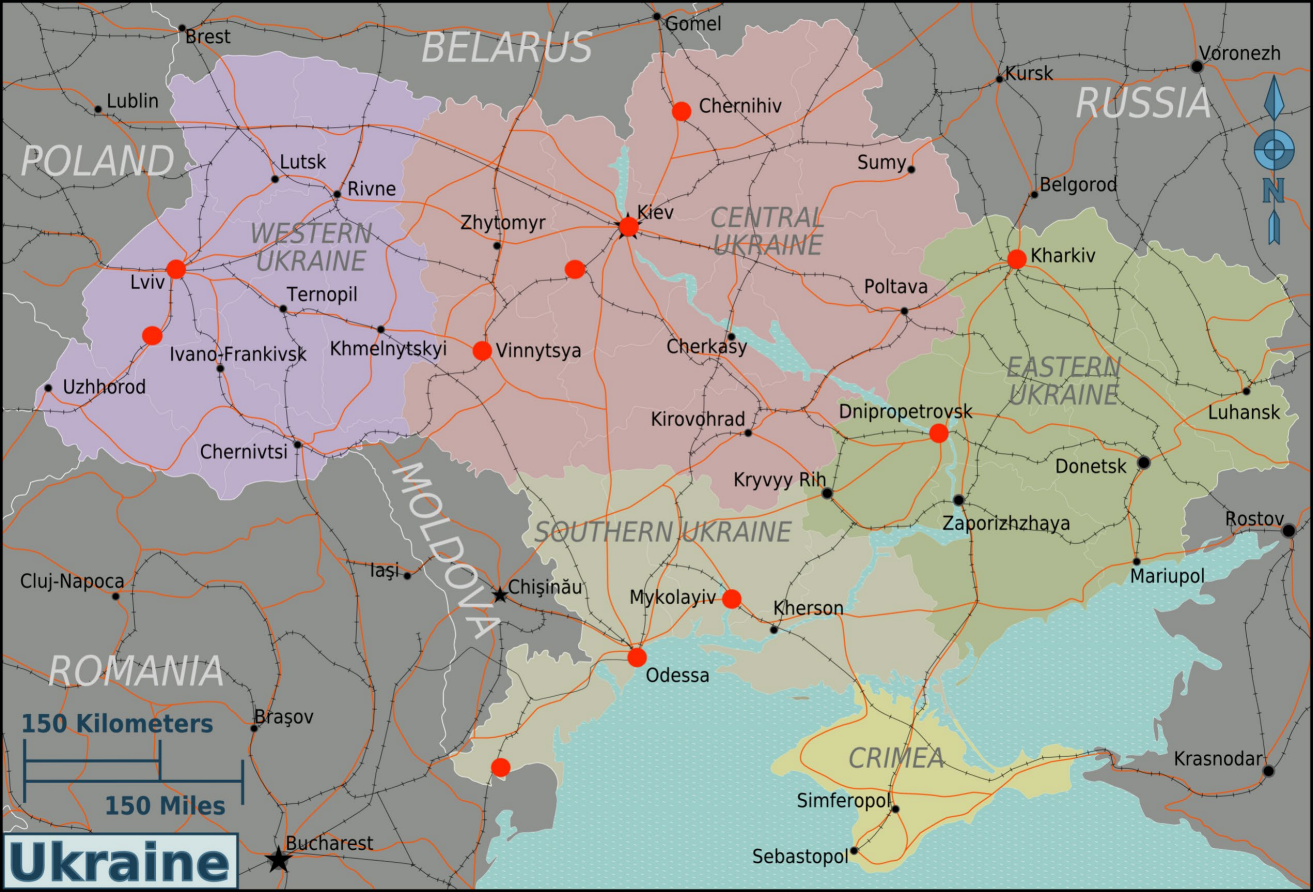
Location of PTF course delivery in 11 cities across Ukraine.

Training sites were identified by International Medical Corps and based on the identified needs of Ukrainian providers who care for pediatric patients. Figure 1 (14) provides a map of locations of all 11 cities where PTF courses were delivered during the program. Course delivery was undertaken at Universities, hotels and hospitals.

### Program evaluation

In alignment with the overarching multi-stream trauma program, the effectiveness of the intervention was assessed through several means. In-person course participation and video access statistics were tracked. Changes in knowledge and self-efficacy were measured individually through pre-and post-course written assessments and self-confidence surveys. Participants completed written evaluations immediately after finishing the course. This information was gathered on paper, transcribed into Kobo Toolbox,^1^ and analyzed with the R Studio statistical package (15). Follow-up evaluations conducted six to eight-weeks post-course measured skill adoption, implementation, and maintenance using participants’ preferred messaging platforms (Telegram, Signal, WhatsApp, or Viber). Knowledge changes were analyzed using paired t-tests, while pre-and post-course self-efficacy surveys were analyzed with McNemar’s test for paired data. Course evaluations included standardized questions about instruction quality, teaching relevance, knowledge gained, and post-course confidence in skills. Handwritten feedback was deidentified, collected in Ukrainian, and translated into English for analysis.

## Results

PTF courses ran from November 2022 to December 2023. A total of 30 PTF courses were taught in the following 11 cities in 8 Oblasts over the total implementation period: Kyiv (*n* = 3), Fastiv (*n* = 1), Dnipro (*n* = 5), Kharkiv (*n* = 3), Chernihiv (*n* = 2), Mykolaiv (*n* = 4), Vinnytsia (*n* = 3), Lviv (*n* = 1), Stryi (*n* = 1), Izmail (*n* = 2), and Odesa (*n* = 5). Overall, a total of 17 unique international instructors were deployed to teach PTF in Ukraine. All instructors underwent a pre-deployment orientation to the PTF course, logistics and safety/security briefing. A total of 446 Ukrainian participants were trained in PTF by international and Ukrainian instructors (85 trained by Ukrainian instructors and 361 by international instructors) and 63 Ukrainian participants completed the ToT course. Demographics of the PTF participants can be found in Table 2.

**Table 2:** Demographics of Ukrainian participants in PTF courses.

Demographics	
Total number of participants	509
Sex	
Female	379 (74.5%)
Male	130 (25.5%)
Age [median (IQR)]	36 (28,48)
Healthcare workers	
Yes	509 (100.0%)
No	0 (0.0%)
Experience	
Years of experience in trauma care (median(IQR))	2 (0,12)
Years of experience in profession (median(IQR))	1 (0,8)
Training/expertise	
Doctor	377 (74.1%)
Nurse	118 (23.2%)
Medical assistant	14 (2.8%)
Specialty	
Pediatrics	125 (24.6%)
Emergency medicine	128 (25.1%)
Family/internal medicine	46 (9.0%)
Anesthesiology	56 (11.0%)
Surgery	86 (16.9%)
Other	35 (6.9%)
Not specified	33 (6.5%)

A 25-question knowledge assessment pre-and post-test was developed to align with overarching PTF course objectives. Participant matched pre-and post-tests demonstrated a significant improvement in knowledge (Table 3). Participant matched 21-question self-confidence and self-efficacy pre-and post-surveys were completed by PTF participants. Variance in total number of participants and number of participants with matched test results were due to multiple reasons including course incompletion by participants and data entry errors resulting in the inability to match participants. Results demonstrated a significant increase in all self-confidence and self-efficacy questions for participants trained by international and Ukrainian instructors. Table 4 demonstrates the aggregate results of all PTF participants. Supplementary Table S1 provides disaggregated tables for each PTF time phase.

**Table 3:** Participant pre-and post-course knowledge assessment, aggregated and stratified by PTF instructor.

PTF Instructor	Participants (*n*=)	Pre-test mean (SD)	Post-test mean (SD)	*p*-value*
International Instructor led courses	310	71.3% (11.5)	86.9% (9.0)	*p* < 0.05
Ukrainian Instructor-led courses	118	73.1% (12.0)	88.4% (9.0)	*p* < 0.05
Total	428	71.8% (11.7)	87.3% (9.0)	*p* < 0.05

*Paired t-test.

**Table 4:** Participant pre-and post-course self-confidence and self-efficacy results (*n* = 351), aggregate of all PTF courses.

Self-confidence and self-efficacy questions	Pre-course	Post-course	*p*-value
I feel comfortable caring for pediatric patients with traumatic injuries	177 (50.4)	307 (87.5)	*p* < 0.001
I feel that I have the skills to provide care for pediatric patients with traumatic injuries	108 (30.8)	313 (89.2)	*p* < 0.001
I feel that I have the knowledge to provide care for pediatric patients with traumatic injuries	125 (35.6)	320 (91.2)	*p* < 0.001
I feel that I understand the ABCDEs of trauma care	204 (58.1)	345 (98.3)	*p* < 0.001
I feel I have an organized approach that allows me to be prepared to care for pediatric trauma patients	199 (56.7)	329 (93.7)	*p* < 0.001
I feel like I have the ability to recognize a critically injured child	246 (70.1)	334 (95.2)	*p* < 0.001
Emergency management of the injured child	97 (27.9)	297 (84.6)	*p* < 0.001
Emergency management of blast injuries in children	53 (15.1)	248 (70.7)	*p* < 0.001
Emergency management of penetrating injuries in children	76 (21.7)	265 (75.5)	*p* < 0.001
Emergency management of blunt trauma in children	109 (31.1)	287 (81.8)	*p* < 0.001
Emergency management of the pediatric patient with shock	106 (30.2)	268 (76.4)	*p* < 0.001
Emergency management of spinal trauma	108 (30.8)	293 (83.5)	*p* < 0.001
Emergency management of the patient with altered mental status	100 (28.5)	269 (76.6)	*p* < 0.001
Emergency management of the patient with difficult breathing	144 (41.0)	296 (84.3)	*p* < 0.001
Emergency management of the pediatric burns	124 (35.3)	302 (89.0)	*p* < 0.001
Emergency management of chemical injuries	61 (17.4)	255 (72.6)	*p* < 0.001
Understanding of Emergency drugs	134 (38.2)	289 (82.3)	*p* < 0.001
Have skills to manage an obstructed blocked airway	104 (29.6)	278 (79.2)	*p* < 0.001
Have skills to manage a patient with difficulty in breathing	129 (36.8)	294 (83.8)	*p* < 0.001
Have skills to manage a patient with bleeding problems	167 (47.6)	318 (90.6)	*p* < 0.001
Have the skills to immobilize injured patients	133 (37.9)	310 (88.3)	*p* < 0.001

A six to eight-week follow up evaluation was sent to course participants via preferred messaging platforms to understand post-course skills utilization and stewardship. Evaluations were sent to all course participants. 91/446 (20.4%) of PTF participants responded. Results of the responses can be found in Supplementary Table S2. Over 73% of PTF participants reported teaching information learned in the course to others including trauma management knowledge and/or procedural skills. When asked if any additional training topics should be taught in the future, over 75% of respondents requested further educational opportunities in pediatric non-trauma emergency care.

Participants filled out immediate post-course evaluations for international instructor-led PTF courses (*n* = 376, 99.5% response rate; Table 5) and the Ukrainian instructor-led PTF courses (*n* = 122, 93.1% response rate; Table 6). While both cohorts received overwhelmingly positive feedback, Ukrainian instructors received higher raw scores across all evaluation points as compared to the international instructors.

**Table 5:** International instructor PTF post-course evaluation forms (*n* = 376).

Question	Yes	Somewhat	No	Missing
The lecture content was customized to the setting that I work or live in	322 (85.6)	50 (13.3)	4 (1.1)	0(0.0)
The skills stations were customized to the setting that I work or live in	303 (80.6)	66 (17.6)	7 (1.9)	0(0.0)
The teaching offered was relevant to me	337 (89.6)	36 (9.6)	3 (0.8)	0(0.0)
The course expanded my clinical knowledge on conditions my patients or community members may have	336 (89.4)	34 (9.0)	6 (1.6)	0(0.0)
The course expanded my clinical practice and assessment skills	333 (88.6)	38 (10.1)	5 (1.3)	0(0.0)
The course was a good balance between instruction and skill practice	335 (89.1)	35 (9.3)	6 (1.6)	0(0.0)
The course was at an appropriate difficulty level for me	321 (85.4)	49 (13.0)	6 (1.6)	0(0.0)
I feel more confident in using specific skills taught during the course as a result of the teaching	335 (89.1)	38 (10.1)	2 (0.5)	1(0.3)

**Table 6:** Ukrainian instructor PTF post-course evaluation forms (*n* = 122).

Question	Yes	Somewhat	No	Missing
The lecture content was customized to the setting that I work or live in	110 (90.2)	12 (9.8)	0 (0.0)	0 (0.0)
The skills stations were customized to the setting that I work or live in	114 (93.4)	8 (6.6)	0 (0.0)	0 (0.0)
The teaching offered was relevant to me	122 (100.0)	0 (0.0)	0 (0.0)	0 (0.0)
The course expanded my clinical knowledge on conditions my patients or community members may have	115 (95.0)	6 (5.0)	0 (0.0)	1 (0.8)
The course expanded my clinical practice and assessment skills	118 (96.7)	4 (3.3)	0 (0.0)	0 (0.0)
The course was a good balance between instruction and skill practice	116 (95.1)	6 (4.9)	0 (0.0)	0 (0.0)
The course was at an appropriate difficulty level for me	112 (91.8)	10 (8.2)	0 (0.0)	0 (0.0)
I feel more confident in using specific skills taught during the course as a result of the teaching	119 (97.5)	3 (2.5)	0 (0.0)	0 (0.0)

## Discussion

During war and conflict, a significant shift in medical care is required to prioritize trauma and acute care injuries (16). This transition involves upskilling or task-shifting healthcare providers to handle the surge of traumatic injuries caused by unique wartime mechanisms of injury and for special populations, including pediatrics (17, 18). Ukraine’s network of pediatric hospitals and academic institutional partners across the country provided a basic infrastructure and setting to undertake a large-scale, country-wide pediatric trauma educational initiative tailored to this population (19). Given the special focus of pediatrics, our unique pediatric trauma fundamentals course provided pediatric-focused education for providers tasked with caring for children during an active war setting in Ukraine. This educational course filled a gap in pediatric trauma education, as other established courses may only briefly address pediatric trauma education in overarching curricula focused on adult emergency and trauma care or focus on providing pediatric trauma education outside of the acute care setting. Furthermore, this course sought to provide a knowledge foundation both for providers with pediatric expertise but no trauma experience, and providers with significant trauma experience but minimal pediatric exposure. During the PTF conceptualization and development, it was clear that the course should be both applicable and tailored to medical providers with specific training and expertise in pediatrics, emergency practitioners, general practitioners and surgeons who may encounter pediatric trauma patients. To accommodate a spectrum of potential learners, the course considered pediatric differences in anatomy, physiology, pathophysiology and common presentations across the pediatrics spectrum of injury.

Over the course of the educational intervention, 509 medical providers were trained in PTF across 11 cities in Ukraine. In addition, this included a cohort of 63 participants in the PTF ToT courses. These participants immediately started to implement independent PTF courses across several regions in Ukraine. The pre−/post-knowledge assessments and self-efficacy surveys demonstrated competency and confidence in participants’ knowledge and their willingness to utilize the skills and knowledge gained during the course. The consistent pre−/post-test knowledge improvement and overwhelmingly positive course feedback for both international and Ukrainian instructors demonstrated a degree of uniformity in the course instructor training and knowledge delivery to participants after transition to fully Ukrainian led instruction. Importantly, evaluation data demonstrated higher raw evaluation scores by Ukrainian instructors as compared to international instructors. This finding suggests the success and importance of the transition to locally taught courses. These findings also suggest other factors, including course delivery in maiden language without interpretation, educational delivery style, and other cultural considerations that may provide more effective course delivery. These findings should be referenced when considering future iterations of PTF across other contexts. Additionally, it is important to note that education is not valuable unless it reaches patient care. Learners reported having already taught information or skills to other medical providers and used those skills in the six to eight-week follow up surveys, indicating that this program is reaching the target population.

There were several limitations to this study. six to eight-week feedback response rate was approximately 20%, likely biased to highly engaged instructors. This provides a limited understanding of how course participants continue to utilize the knowledge gained from PTF. Given the breadth and length of the intervention, 17 international instructors were required to undertake this intervention. This included instructors with backgrounds in pediatric emergency medicine, pediatric and general surgery, and adult emergency medicine. To mitigate variations in teaching content and quality of teaching, a detailed, point-by-point international instructor manual was provided to all instructors and discussed in depth during the pre-departure orientation. For those instructors with significant trauma experience but limited pediatric experience, a pediatric-specific pre-departure orientation was provided in addition to the required pre-departure orientation. Additional challenges to the standardization of classes included the risk of active conflict affecting course delivery. This reality included interruptions of classes due to air raid sirens, requiring courses to be held in bomb shelters, basements, or parking garages due to periods of high threat, and unmeasured psycho-social stressors that are ever present in a war-time society. Risks to personnel were mitigated through safety and security protocols and risk assessments by our partner organization, International Medical Corps. Despite these disruptive forces, monitoring and evaluation of the courses consistently demonstrated improvement in knowledge and skills and uniformity of classes over the course of the longitudinal intervention.

## Conclusion

The PTF educational initiative demonstrates a successful three-phase model for implementing an educational initiative for providers caring for children in active conflict zones. Despite the safety and security challenges, this model also demonstrates the value of an academic/non-governmental organization partnership to help mitigate risks through safety and security preparation, planning, and real time risk mitigation in an active conflict zone such as Ukraine. Ukrainian instructors provide course experiences similar or superior to international instructors, likely due to multiple factors related to language, culture and context. Finally, building partnerships between academic institutions is a proven and promising model for sustainability and localization of long-term training programs.

## Data Availability

The raw data supporting the conclusions of this article will be made available by the authors, without undue reservation.

## Footnotes

^1^https://www.kobotoolbox.org/
