# Supplementary material for: Expanding trauma education during war: pediatric trauma fundamentals training in Ukraine
Source: Front Public Health. 2024 Sep 6;12:1448075. doi: 10.3389/fpubh.2024.1448075 (PMC11413807; doi:10.3389/fpubh.2024.1448075)
Supplement: Supplementary file 1 [file Table_1.DOCX]

Supplement 1. Self-efficacy analysis, disaggregated by instructor group

| International instructor-led course |  |  |  |
| --- | --- | --- | --- |
|  | n=234 | | |
| Question: | Pre-course | Post-course | p-value |
| *Participants who agree or strongly agree to the following (4-point Likert scale):* | | | |
| I feel comfortable caring for pediatric patients with traumatic injuries | 120 (51.3%) | 208 (88.9%) | p < 0.001 |
| I feel that I have the skills to provide care for pediatric patients with traumatic injuries | 78 (33.3%) | 214 (91.5%) | p < 0.001 |
| I feel that I have the knowledge to provide care for pediatric patients with traumatic injuries | 84 (35.9%) | 216 (92.3%) | p < 0.001 |
| I feel that I understand the abcde s of trauma care | 129 (55.1%) | 228 (97.4%) | p < 0.001 |
| I feel I have an organized approach that allows me to be prepared to care for pediatric trauma patients | 133 (56.8%) | 223 (95.3%) | p < 0.001 |
| I feel like I have the ability to recognize a critically injured child | 168 (71.8%) | 219 (93.6%) | p < 0.001 |
| *Participants who were confident or very confident in the following (4-point Likert scale):* | | | |
| Emergency management of the injured child | 66 (28.2%) | 203 (86.7%) | p < 0.001 |
| Emergency management of blast injuries in children | 37 (15.8%) | 167 (71.4%) | p < 0.001 |
| Emergency management of penetrating injuries in children | 52 (22.2%) | 177 (75.6%) | p < 0.001 |
| Emergency management of blunt trauma in children | 77 (32.9%) | 191 (81.6%) | p < 0.001 |
| Emergency management of the pediatric patient with shock | 74 (31.6%) | 182 (77.8%) | p < 0.001 |
| Emergency management of spinal trauma | 80 (34.2%) | 197 (84.2%) | p < 0.001 |
| Emergency management of the patient with altered mental status | 71 (30.3%) | 179 (76.5%) | p < 0.001 |
| Emergency management of the patient with difficult breathing | 102 (43.6%) | 201 (85.9%) | p < 0.001 |
| Emergency management of the pediatric burns | 90 (38.5%) | 203 (86.8%) | p < 0.001 |
| Emergency management of chemical injuries | 41 (17.5%) | 175 (74.8%) | p < 0.001 |
| Understanding of Emergency drugs | 94 (40.2%) | 194 (82.9%) | p < 0.001 |
| Have skills to manage an obstructed blocked airway | 74 (31.6%) | 187 (79.9%) | p < 0.001 |
| Have skills to manage a patient with difficulty in breathing | 92 (39.3%) | 199 (85.0) | p < 0.001 |
| Have skills to manage a patient with bleeding problems | 120 (51.3%) | 212 (90.6%) | p < 0.001 |
| Have the skills to immobilize injured patients | 92 (39.3%) | 211 (90.2%) | p < 0.001 |

| Ukrainian instructor-led course |  |  |  |
| --- | --- | --- | --- |
|  | n=117 | | |
| Question: | Pre-course | Post-course | p-value |
| *Participants who agree or strongly agree to the following (4-point Likert scale):* | | | |
| I feel comfortable caring for pediatric patients with traumatic injuries | 57 (48.7%) | 99 (84.6%) | p < 0.001 |
| I feel that I have the skills to provide care for pediatric patients with traumatic injuries | 30 (25.6%) | 99 (84.6%) | p < 0.001 |
| I feel that I have the knowledge to provide care for pediatric patients with traumatic injuries | 41 (35.0%) | 104 (88.9%) | p < 0.001 |
| I feel that I understand the abcde s of trauma care | 75 (64.1%) | 117 (100%) | p < 0.001 |
| I feel I have an organized approach that allows me to be prepared to care for pediatric trauma patients | 66 (56.4%) | 106 (90.6%) | p < 0.001 |
| I feel like I have the ability to recognize a critically injured child | 78 (66.7%) | 115 (98.3%) | p < 0.001 |
| *Participants who were confident or very confident in the following (4-point Likert scale):* | | | |
| Emergency management of the injured child | 32 (27.4%) | 94 (80.3%) | p < 0.001 |
| Emergency management of blast injuries in children | 16 (13.7%) | 81 (69.2%) | p < 0.001 |
| Emergency management of penetrating injuries in children | 24 (20.5%) | 88 (75.2%) | p < 0.001 |
| Emergency management of blunt trauma in children | 32 (27.4%) | 96 (82.1%) | p < 0.001 |
| Emergency management of the pediatric patient with shock | 32 (27.4%) | 86 (73.5%) | p < 0.001 |
| Emergency management of spinal trauma | 28 (23.9%) | 96 (82.1%) | p < 0.001 |
| Emergency management of the patient with altered mental status | 29 (24.8%) | 90 (76.9%) | p < 0.001 |
| Emergency management of the patient with difficult breathing | 42 (35.9%) | 95 (81.2%) | p < 0.001 |
| Emergency management of the pediatric burns | 34 (29.1%) | 99 (84.6%) | p < 0.001 |
| Emergency management of chemical injuries | 20 (17.1%) | 80 (68.4%) | p < 0.001 |
| Understanding of Emergency drugs | 40 (34.2%) | 95 (81.2%) | p < 0.001 |
| Have skills to manage an obstructed blocked airway | 30 (25.6%) | 91 (77.8%) | p < 0.001 |
| Have skills to manage a patient with difficulty in breathing | 37 (31.6%) | 95 (81.2%) | p < 0.001 |
| Have skills to manage a patient with bleeding problems | 47 (40.2%) | 106 (90.6%) | p < 0.001 |
| Have the skills to immobilize injured patients | 41 (35.0%) | 99 (84.6%) | p < 0.001 |
