# Supplementary material for: Expanding trauma education during war: pediatric trauma fundamentals training in Ukraine
Source: Front Public Health. 2024 Sep 6;12:1448075. doi: 10.3389/fpubh.2024.1448075 (PMC11413807; doi:10.3389/fpubh.2024.1448075)
Supplement: Supplementary file 2 [file Table_2.DOCX]

Supplement 2. Six to eight week follow up survey results

|  | (n=91) |
| --- | --- |
| Question | Percentage of Respondents (n, (%)) |
| 1. Do you feel that the training has had (or will have) a life-saving effect in your management of a pediatric trauma patient? |  |
| Yes | 89 (97.8 ) |
| No | 11 (2.2 ) |
|  |  |
| 2. How was the training helpful to you? (Select all that apply) |  |
| I Learned new knowledge and/or facts about pediatric trauma | 78 (85.7) |
| I learned new procedures applicable to my practice | 65 (71.4 ) |
| I now feel more confident to care for a pediatric trauma patient | 65 (71.4 ) |
| The training improved my management of a trauma patient. | 59 (64.8 ) |
| The training was not helpful to me. | 1 (1.1) |
|  |  |
| 3. How have you applied the pediatric trauma training in patient care?  (Select all that apply) |  |
| Patient management (plans of care, medications used) | 52 (57.1) |
| Performed a new skill that I learned in the course | 57 (62.6 ) |
| Used equipment in my unit that I previously did not know how to use | 30 (33.0) |
| Other way I applied | 15 (16.5) |
| No, I have not integrated the training into my practice. | 3 (3.3) |
|  |  |
| 4. Have you taught information that you learned in the course to others? (Select all that apply) |  |
| Yes, I taught others how to perform procedures learned in the course | 43 (47.3) |
| Yes, I taught others trauma management knowledge/information | 67 (73.6) |
| No, I have not taught this information to others. | 10 (11.0) |
|  |  |
| 5. What educational opportunities would you like to see in the future? (Select all that apply) |  |
| Pediatric emergency care (non-trauma) | 69 (75.8) |
| Skill refresher: placing IVs, urinary catheters, NG/OG tubes, securing ET tube | 44 (48.4) |
| More advanced/higher level knowledge in trauma care | 42 (46.2) |
| Basic adult trauma care training | 33 (36.3) |
| Trauma care for special populations (geriatric, pregnancy) | 32 (35.2) |
| Additional procedural skills | 41 (45.2) |
| Sedation for procedures outside of the operating room | 28 (30.8) |
| More war-specific trauma training (blast injury, penetrating trauma, advanced burn care) | 51 (56.0) |
| Psychological first aid | 47 (51.7) |
| Triage training | 37 (40.7) |
| Mass Casualty response | 39 (42.9) |
| Caring for victims of chemical, biological, radiologic, nuclear and explosive injuries | 35 (38.5) |
| Caring for victims of sexual violence | 29 (31.9) |
| Forensic principles for potential war crimes (documentation, evidence collection) | 21 (23.1) |
| Intubation and advanced respiratory support | 41 (45.2) |
| Point-of-care Ultrasound | 40 (44.0) |
| Other | 4 (4.4) |
| None of the above | 0 (0.0) |
